# Cognitive Mechanisms Underlying Memory Advantages in Bridge Experts: Based on Suit Categorization and Honor Card Rules

**DOI:** 10.3390/bs15020125

**Published:** 2025-01-24

**Authors:** Yanzhe Liu, Yan Liu, Bingjie Zhao, Qihan Zhang

**Affiliations:** 1 Key Research Base of Humanities and Social Sciences of the Ministry of Education, Academy of Psychology and Behavior, Tianjin Normal University, Tianjin 300387, China; liuyanzhe040618@163.com (Y.L.);; 2 Faculty of Psychology, Tianjin Normal University, Tianjin 300387, China; 3 School of Sports Science and Physical Education, Tianjin Normal University, Tianjin 300387, China

**Keywords:** bridge experts, memory, abstract rule, visual familiarity

## Abstract

To explore the memory advantage and the underlying mechanisms of bridge experts, this study conducted two experiments. Experiment 1 investigated the effects of the suit categorization rule and the rank ordering rule on the memory performance of bridge experts when memorizing hands. The findings revealed that the suit categorization rule significantly influenced the memory advantage of bridge experts, regardless of whether the task involved recognition or free recall. Conversely, the rank ordering rule had no discernible effect on their memory performance, though the honor card information within this rule notably impacted their memory. Building on the first experiment, Experiment 2 further examined the roles of visual familiarity induced by the suit categorization and honor card rules, alongside the abstract knowledge embedded in these rules, on the memory performance of bridge experts. The results demonstrated that visual familiarity influenced recognition among bridge experts, while both visual familiarity and abstract knowledge jointly contributed to recall performance. These research findings concurrently support both chunking/template theory and SEEK theory.

## 1. Introduction

Bridge, as a card game renowned for its emphasis on strategy, cooperation, and memory skills (World Bridge Federation, 2024b; Punch & Snellgrove, 2021), has garnered a global following. The data disclosed by the World Bridge Federation reveals a substantial presence of approximately 1 million bridge players distributed across 116 countries (World Bridge Federation, 2024a), with an estimated global population of enthusiasts exceeding 220 million (Andrew Robson Bridge Club, 2024). Previous research has identified that bridge training confers cognitive benefits, particularly in memory and perception processes (Hen-Herbst et al., 2023; Zhang, 2022), effectively delaying the onset of age-related cognitive decline (Małysa, 2020; Briggs, 2000; L. C. Smith & Hartley, 1990), thus playing a significant role in prevention of dementia (Małysa, 2020).

The expert–novice paradigm emerges as a robust approach for investigating the cognitive plasticity induced by athletic training, such as in the case of bridge (Ballesteros et al., 2024; De Winter et al., 2023; Yin et al., 2021; Bai et al., 2020; Bilalić et al., 2010; Campitelli et al., 2005). Previous studies have found that, compared to novices or laypersons, bridge experts exhibit a significant advantage in memory performance, especially when dealing with familiar hand materials (Briggs, 2000; L. C. Smith & Hartley, 1990). For instance, Engle and Bukstel (1978) devised two distinct categories of hand materials: structured hands and unstructured hands. Structured hands involve playing cards categorized by suit into four columns, with each column arranged in order of card rank, whereas unstructured hands consist of randomly presented cards without any arrangement based on suit or rank. The results indicate that bridge experts exhibit superior memory performance when dealing with structured hands, as compared to novices or laypersons. Similarly, Charness (1979) corroborated this observation, highlighting the memory superiority of bridge experts in structured hands. Due to various factors, including the intricate nature of bridge, we recently conducted searches on academic websites such as Web of Science and Google Scholar using carefully selected keywords such as “Bridge”, “Expert”, “Memory”, and “Rule”. Our search yielded a limited number of relevant studies, with only two articles specifically addressing the memory advantages observed in bridge experts (Charness, 1979; Engle & Bukstel, 1978). However, these studies have several limitations and do not yet elucidate the underlying mechanisms responsible for the memory advantages exhibited by bridge experts: (1) Due to the limited sample size, the reliability and external validity of the results require further validation; (2) Structured hands in bridge encompass a plethora of visual information and bridge rules, rendering it challenging to discern which information aids experts’ memory; (3) Controversy exists regarding the performance of experts on unstructured hands, with limited insights provided by Engle and Bukstel (1978) in comparing expert and novice memory performance on unstructured hands, while Charness (1979) found no significant differences between the two. The memory advantage of bridge experts, therefore, necessitates additional validation and warrants further investigation into its underlying causes. 

The games of bridge and chess both fall under the mind sports. The definition of mind sports is that players need to apply learned rules or procedures to accomplish cognitive tasks (Zhao et al., 2022). Consequently, the studies and theories elucidating the underlying mechanisms behind memory advantages in chess experts can offer valuable insights into understanding the memory advantages observed in bridge experts. The chunking theory posits that the memory advantage in chess experts arises from a vast repository of chunks stored in long-term memory, which primarily consist of specific pieces distributed across particular board positions, such as fixed chess configurations (Gobet & Clarkson, 2004; Chase & Simon, 1973a). De Groot’s seminal research required subjects to engage in immediate free recall after memorizing chess positions. The results demonstrated that chess masters exhibit superior search efficiency and recall of chess positions compared to players of lower proficiency (De Groot, 1946, 1965/1966). Subsequently, Chase and Simon (1973a, 1973b), through repeated experiments, found that, compared to novices, chess experts recall larger and more numerous chunks of the chessboard, showing significantly better memory performance; this led to the formulation of the renowned chunking theory. Based on this, Gobet and Simon (1998, 1996b) proposed the template theory, suggesting that chess experts store templates in their long-term memory rather than merely chunks. These templates consist of chunk-like structures with slots for information, encompassing not only the aforementioned chunks but also abstract schematics such as the movement of pieces (Gobet, 1998; Gobet & Simon, 1996b). The formation of these chunks or templates heavily relies on experts’ repeated visual discernment of stimuli, thereby making the visual familiarity of memory materials a significant determinant of experts’ memory performance. Prior research involving recognition and free recall tasks has shown that, compared to random chess configurations, experts exhibit enhanced memory performance when confronted with genuine chess configurations (Gong, 2015; Campitelli et al., 2007; Chase & Simon, 1973a); Moreover, experts exhibit memory advantages for chess materials compared to newly defined patterns (E. T. Smith et al., 2021; Campitelli et al., 2007). However, experts’ memory performance is also influenced by higher-level conceptual knowledge and semantic information (Cooke et al., 1993), with highly skilled players possessing a greater amount of extensively conceptualized knowledge (Gobet & Waters, 2023; Chassy & Gobet, 2011). The SEEK theory posits that the memory advantage of chess experts stems from more abstract and highly generalized knowledge stored in long-term memory rather than specific visual–spatial information (Holding, 1985). Huang et al. (2023) found that expertise-related memory advantages are closely linked to the acquisition of abstract rule knowledge, such as piece movements. Gobet and Waters (2023) further demonstrated that a profound mastery of abstract rule knowledge pertaining to the interactions between chess pieces also contributes significantly to memory advantages. It is evident that there is still some debate regarding the underlying mechanisms elucidating the memory advantages exhibited by chess experts. Further investigation is warranted to examine the memory benefits observed in bridge experts based on these findings. Furthermore, the LTWM theory posits that experts’ memory capabilities derive from an encoding system and retrieval mechanisms deeply embedded in long-term memory. Experts transcend the limitations of short-term memory capacity; their memory prowess is intimately linked to their extensive knowledge bases and profound experience. Consequently, experts exhibit remarkable memory abilities (Ericsson & Kintsch, 1995). They utilize relevant knowledge structures within their LTWM as cohesive information units, which significantly enhances their effective working memory capacity (Guida et al., 2009). The aforementioned theories collectively suggest that the memory advantage of experts stems from their rich knowledge structures stored in long-term memory, which extend their working memory capacity (Guida et al., 2009). Consequently, experts are capable of transferring more information to long-term memory or maintaining it in short-term memory for extended periods, thereby demonstrating a significant memory advantage.

Bridge and chess differ significantly in their gameplay rules. Compared to chess, which emphasizes the spatial relationships of pieces, bridge focuses more on comparing cards based on suit and rank (Briggs, 2000; Charness, 1987, 1983, 1979; Engle & Bukstel, 1978). The gameplay of bridge can be divided into two phases: (1) Bidding phase: Players communicate information regarding the suits and values of their hands to their partner through a bidding system, wherein the hand’s worth is primarily determined by the presence of honor cards and HCP (high card points), with A, K, Q, J, and 10 corresponding to 4, 3, 2, 1, and 0 HCP, respectively (Charness, 1987, 1983). (2) Playing phase: Bridge players determine the outcome of each trick by comparing the ranks of cards within the same suit, with higher-ranked cards possessing a greater likelihood of winning a trick (World Bridge Federation, 2024b; Briggs, 2000). According to the regulations of bridge, players need to arrange their hands in a specific sequence during gameplay in order to facilitate the computation of hand valuation (World Bridge Federation, 2024b). The structuring of hands involves the utilization of ordering rules, which can be classified into two main types: the suit categorization rule and the rank ordering rule. The former entails classifying their hands according to suit, while the latter involves sorting the cards within each suit in descending order based on their ranks. Previous studies have demonstrated that the memory advantage observed in bridge experts is contingent upon their adept utilization of these two rules, as they exhibit superior performance in recalling structured hands based on said rule (Charness, 1979; Engle & Bukstel, 1978). It is evident that these two rules play a crucial role in the memory advantage of bridge experts. However, previous studies have frequently amalgamated these two rules, examining their collective impact on the memory advantage of bridge experts. The singular effects of these rules on expert memory advantage have yet to be clarified, and the interaction between these two rules remains undisclosed. Moreover, the rank ordering rule in bridge carries a dual significance, encompassing both the numerical value of the card and its HCP value. As previously mentioned, a card’s value is determined by its HCP, with honor card rules defining the correspondence between card ranks and HCP. The previous literature on strategies employed by bridge experts has shown that memorizing honor cards is the most commonly utilized memory strategy among these experts (THE BRIDGE WORLD, 2024; Charness, 1979; Engle & Bukstel, 1978). Therefore, when examining the impact of the rank ordering rule on the memory advantages of bridge experts, it is crucial to consider the role played by honor cards carrying HCP. Moreover, the prolonged utilization of these rules not only enhances experts’ visual familiarity with the cards (Bouts & Van Avermaet, 1992; Charness, 1979) but also internalizes the abstract knowledge embedded within the rules into their cognitive schemata (Küchelmann et al., 2022; Holding, 2021; Simões et al., 2020). According to the chunking/template theory, individuals with a stronger visual familiarity of stimulus materials demonstrate enhanced memory performance, whereas the SEEK theory posits that the greater the mastery of abstract knowledge of the rules, the more pronounced the enhancement in memory performance. Therefore, further investigation is warranted to explore the impact of these two factors on the memory advantage observed in bridge experts.

Furthermore, exploring the intrinsic mechanisms and the potential transferability of memory advantages among bridge players holds potential significance for the training and development of bridge athletes. This study can extend the findings of existing research on chess and other intellectual sports, providing more direct data and empirical support for studies on memory advantages among experts. This could enhance our understanding of cognitive enhancements through game-based training and potentially influence training methodologies across various intellectual sports. By deepening our comprehension of how strategic memory use in bridge can be generalized to other cognitive domains, our research may also offer valuable insights into the broader implications of specialized training practices.

To elucidate the underlying mechanisms of memory advantage in bridge experts, this research conducted two experiments. In light of prior studies that utilized recognition and free recall tasks (Gong, 2015; Campitelli et al., 2007; Chase & Simon, 1973a), which revealed inconsistent memory contents, our experiments consequently adopted these two types of memory tasks. Specifically, the recognition task requires participants to recall after a one-minute interval in order to evaluate long-term memory (Wickelgren, 1973). The recall task required participants to immediately retrieve information after a five-second delay to explore working memory (Chase & Ericsson, 1982). Experiment 1 employed an expert–novice paradigm, manipulating the sorting methods of each card in the hand (i.e., whether they were presented according to the hand suit categorization rule and hand rank ordering rule) to investigate the effects of these rules on the memory performance of bridge experts. The memory performance for honor cards was also investigated, taking into account the significant role demonstrated by Charness’s research (Charness, 1987, 1983). Based on the studies conducted by Engle and Bukstel (1978) as well as Charness (1979), it is established that employing structured hands constructed according to these two rules confers greater advantages for expert memory. Furthermore, considering the gameplay characteristics of bridge, suit categorization and rank ordering occur simultaneously during hand organization, suggesting that the simultaneous influence of these two rules on cognitive processing is a possibility. Therefore, we proposed Hypothesis 1: Both the suit categorization rule and the rank ordering rule influence expert memory performance and exhibit a close relationship. Under structured hand conditions, when hands satisfy both of these rules simultaneously, bridge experts demonstrate superior memory performance compared to novices; however, under unstructured hand conditions where hands do not meet both of these rules, there is no significant difference in memory performance between bridge experts and laypersons. Experiment 2, building upon the first one, further investigated the role of the specificity information (visual familiarity induced by rules) and abstract information (abstract knowledge within the rules) in the memory performance of bridge experts by modifying the objects or breaking the abstract knowledge within the rules. Drawing from the advancements in chunking/template theory, SEEK theory, and the research by Holding (2021), it is postulated that both specific and abstract information about the rules may impact experts’ memory advantage. Hence, we proposed Hypothesis 2: The alteration of the object of the rules, irrespective of the conditions, will disrupt the established visual familiarity based on those rules, thereby significantly impacting the expert’s memory performance. The utilization of uncorrupted abstract rule knowledge, however, facilitates the enhancement of memory performance for the bridge expert in the event of a change in the rule object.

## 2. Experiment 1: The Influence of the Suit Categorization Rule and the Rank Ordering Rule on the Memory Advantage of Bridge Experts

### 2.1. Methods

#### 2.1.1. Participants

Bridge experts (referred to as “experts” hereafter) were recruited nationwide in China according to the following criteria: (1) Experienced contract bridge players with a minimum of 10 years’ proficiency in the game (Trevisan et al., 2022; Ericsson et al., 1993; Simon & Chase, 1973); (2) Bridge training duration exceeding 10,000 h (Toner et al., 2021; Lordo, 2021; Berlot et al., 2020; Simon & Chase, 1973); (3) Reaching the level of National Master or higher in bridge. The “Technical Level Standards for Members of the Chinese Contract Bridge Association” (Chinese Contract Bridge Association, 2023) specify that a Bridge National Master must possess a minimum of 250 master points, including at least 5 platinum or gold points, 30 silver points, and 25 red points. To acquire platinum or gold points, players are required to achieve a top-eight ranking in the highest-level national bridge tournament.

This experiment recruited a total of 52 experts (expert group) and 55 laypersons (control group). There were no significant differences observed between the two groups in terms of gender, age, educational background, or intelligence, as shown in Table 1. All participants in the control group were familiar with playing cards but completely unfamiliar with bridge. In addition, the expert group had bridge experience ranging from 10 to 30 years. They underwent weekly bridge training for an average of 18.15 (*SD* = 4.91) hours, with a total training time ranging from 10,018.29 to 31,307.14 h. Their master points ranged from 250 to 7668. According to the “Technical Level Standards for Members of the Chinese Contract Bridge Association” for classifying expert technical levels, there are 41 National Masters, 8 Life Masters, and 3 Grand Masters. This experiment was approved by the Ethics Committee of Tianjin Normal University (No. 2023112302). Prior to the formal experiment, all participants signed informed consent forms, and after the experiment, they received a certain amount of monetary compensation.

#### 2.1.2. Experimental Design

This experiment employed two types of memory tasks, recognition and recall, to investigate the underlying mechanisms behind the memory advantage in bridge experts. The experimental design for these two tasks consists of a three-factor mixed experimental design, with 2 (groups: expert/EG, control/CG) × 2 (hand suit categorization: regular/CR, irregular/CI) × 2 (hand rank ordering: regular/OR, irregular/OI). Group is a between-subject variable, while hand suit categorization and hand rank ordering are within-subject variables. In the recognition task, the dependent variables mainly include the subjects’ accuracy in the recognition task and their reaction time for correct recognition. In the recall task, the dependent variable is mainly the subjects’ accuracy in recalling cards, i.e., the proportion of correctly recalled cards to the total number of a hand.

#### 2.1.3. Experimental Questionnaire

The Raven’s Progressive Matrices: This test is typically understood as a measure of nonverbal reasoning and is widely employed by various experts for intelligence assessment due to its strong reliability and validity (Paz-Baruch et al., 2022; Y. Wang et al., 2020; Burgoyne et al., 2016; Unterrainer et al., 2011; L. C. Smith & Hartley, 1990). The test consists of 60 graphic reasoning questions, divided into 5 difficulty levels, with 12 questions at each level. In this research, the test was presented through the online platform TClab (https://www.testcloudlab.com/). The title and options of each question were presented sequentially on the screen. Participants were required to fill in the missing parts in the pictures by pressing corresponding numbers on the keyboard, and the next question was presented after pressing the key until 15 min of answering time was up. Each correct answer scores 1 point, while incorrect answers do not score.

Post-experimental questionnaire: To understand the memory strategies employed by participants in the recognition and recall tasks, we designed a post-experimental questionnaire consisting of the following question: “What strategy did you use to memorize the hands in the recognition/recall task just now?” Participants were asked to either type or verbally inform the experimenter.

#### 2.1.4. Experimental Materials

**Figure 1:**
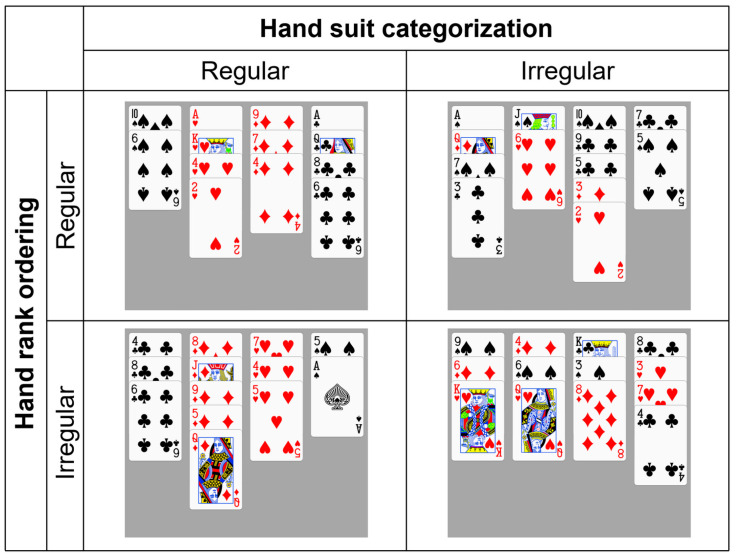
Materials for Experiment 1.

The experimental materials for both the recognition and recall tasks consist of self-made hand materials with a gray background. Each hand material is composed of 13 playing cards divided into 4 columns. The hand materials were created using PowerPoint, with dimensions of 1335 (width) × 1553 (height) pixels. In each set of hand materials, the presentation of the 13 playing cards follows four conditions: (1) Regular suit categorization and rank ordering (CR-OR): the 13 playing cards are categorized by suit into four columns, each column ordered by card rank, also referred to as structured hands; (2) Regular suit categorization and irregular rank ordering (CR-OI): the 13 playing cards are categorized by suit into four columns, but the rank ordering of cards in each column is random; (3) Irregular suit categorization and regular rank ordering (CI-OR): the suits of cards in each column are randomly assigned, but each column ordered by card rank; (4) Irregular suit categorization and rank ordering (CI-OI): the suit categorization and rank ordering of cards in each column are random, also referred to as unstructured hands. As shown in Figure 1, the quantities of suits, honor cards (A, K, Q, J, 10), honor cards with HCP (A, K, Q, J), and card “10” were controlled and balanced across all four experimental conditions in both recognition and recall tasks. Additionally, the HCP values and suit patterns of each hand were also standardized. Specifically, there are no significant differences in the quantities of spades, hearts, diamonds, and clubs across these four conditions. On average, the hands of each condition contain five honor cards, with four having HCPs, one “10” card, and a total of ten HCPs. The suit patterns used were the ten most common in bridge (4432, 5332, 5431, 4333, 5422, 6331, 6322, 6421, 5521, 4441). According to the probability tables published by BridgeHands (2011), these ten suit patterns collectively account for a high proportion (91.1%) of occurrences and do not significantly influence memory due to the absence or excessive presence of any particular suit. Under the condition of regular hand suit categorization, the suits in each column are balanced according to the Latin Square design principle. The specific parameters for the experimental materials can be found in the Supplementary Materials.

#### 2.1.5. Experimental Tasks

**Figure 2:**
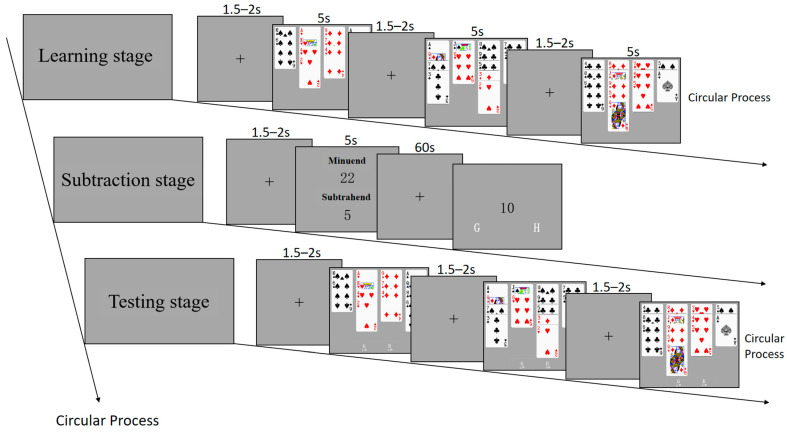
Recognition task procedure.

Recognition task: Participants were instructed to acquire and memorize a set of hand materials, followed by the subsequent test to determine their previous learning. This task consisted of three stages: learning, subtraction, and testing. During the learning stage, participants were directed to memorize hand materials displayed on the screen. A total of 80 materials were presented, with 20 materials per condition. Each material was shown for 5 s, interspersed with a fixation point lasting for 1.5–2 s between each material. During the subtraction stage, participants were instructed to consecutively perform subtraction calculations. A minuend and consecutive subtrahend were presented at the center of the screen. Subsequently, a numerical value was presented at a specific interval, and participants were required to judge whether this number appeared in their mental arithmetic process. In the testing stage, participants were instructed to determine which of the presented materials they had previously learned and which they had not. The testing stage consists of 60 hand materials, each displayed for a maximum of 5 s and disappearing after the judgment key was pressed. A fixation point was presented for 1.5–2 s between materials. Half of the hand materials in the testing stage were consistent with those in the learning stage (with basic balance across four conditions), 1/6 had a change in the HCP of a single honor card (e.g., from spade K to spade J), 1/6 had a change in the suit of a single honor card (e.g., from spade K to heart K), and the remaining 1/6 consisted entirely of new hand materials that are different from those in the learning stage. The suit patterns of these unlearned hand materials in the testing stage did not exceed the range of the ten common suit patterns mentioned above. These three stages were evenly distributed across four blocks, with each block consisting of learning 20 hand materials, performing continuous subtraction tasks for 1 min, and making recognition judgments for 15 hand materials during the testing stage. The task flow is illustrated in Figure 2. The order of the blocks was randomized, and the rest time between blocks was determined by the participants. The key responses during the subtraction and testing stages were balanced among participants: Half of them responded “learned/seen” by pressing the G key, while the other half responded with the H key, and vice versa.

**Figure 3:**
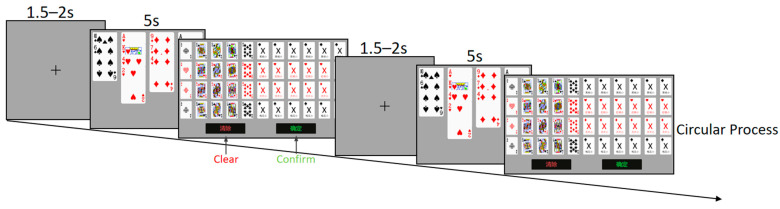
Recall task procedure.

Recall task: Participants are required to recall and reconstruct the hand they have previously learned. The task consists of a total of 40 hand materials, with 10 materials under each condition, different from those used in the recognition task. According to research, bridge experts employ relatively stable memory strategies, focusing on remembering honor cards (THE BRIDGE WORLD, 2024; Charness, 1979; Engle & Bukstel, 1978). To prevent differences between experts and novices caused by these memory strategies, participants in this research were instructed to memorize cards ranked lower than 10 as “X” prior to the formal experiment. At the beginning of each trial, a fixation point is presented for 1.5–2 s, followed by the random presentation of hand materials (5 s). After the hand materials disappear, participants are immediately prompted to use the mouse to select the playing cards they just remembered on the response screen. There is no time limit for this process, and participants can modify or clear their selections by clicking the “Clear” button. After completing all recalls, participants need to click the “Confirm” button, as shown in Figure 3. This process is repeated 40 times until the recall task ends.

#### 2.1.6. Experimental Procedure

This experiment utilizes the TClab online experimental platform (https://www.testcloudlab.com/). The experimental program is self-developed. Once published online, participants are expected to use a computer to complete the experimental tasks. The specific procedure is outlined below:

(1) Fill out the informed consent form and complete the demographic information survey. The control group is required to provide their age, educational background, gender, and other relevant details. In addition to these specifics, participants in the expert group should also provide information about their years of experience in playing bridge, master points earned, technical level achieved, and weekly practice time dedicated to bridge.

(2) To eliminate the impact of individual differences in key press response on the results, both the expert group and control group must complete a color discrimination reaction task before the formal experiment begins. Specifically, a colored circle will be displayed on the screen for 1 s, requiring participants to quickly respond with a key press according to the color of the circle: half of them press the G key for green and the H key for red, while the other half do the opposite. Following each response, a fixation point appears for 1–1.5 s. Green and red circles each appeared ten times during this task.

(3) Once participants have fully practiced the experimental tasks, the formal experiment is conducted. First, participants complete the recognition task, followed by the recall task. After each task, participants are required to fill out a self-developed post-experiment questionnaire. Participants are provided with sufficient rest between the two tasks.

#### 2.1.7. Data Analysis

Data processing: Experimental data were processed according to the following criteria: (1) Extreme and invalid data were removed, including data from participants in both the expert and control groups whose task performance exceeded or fell below three standard deviations from the mean, as well as data from participants who terminated the experiment prematurely. (2) Data from participants with an accuracy below 75% in the consecutive subtraction stage were excluded.

To eliminate the interference of key response on experimental results, independent-sample *t*-tests were conducted on the accuracy and reaction time of both the expert and control groups in the discrimination reaction task. If significant differences were found, they would be included as covariates in the subsequent analysis of the recognition task.

Recognition task: (1) Analysis of participants’ recognition performance. The recognition accuracy and correct recognition reaction time of the participants were analyzed using a mixed-design analysis of variance (ANOVA), with factors including groups (expert, control), hand suit categorization (regular, irregular), and hand rank ordering (regular, irregular). (2) Analysis of the expert group’s rejection performance of unlearned materials. The unlearned hand materials in the recognition task testing stage primarily consist of two components: hands with slight variations, where only one honor card’s suit or HCP is changed (similar condition), and completely new hands that differ from those encountered during the learning stage (different condition). Analyzing the expert group’s correct rejection rate of these unlearned materials helps reveal the accuracy of expert memory under diverse conditions. Consequently, pairwise comparisons were conducted between the expert group’s correct rejection rates for unlearned hand materials or compared against a random probability of 0.5.

Recall task: (1) Analysis of participants’ recall performance. The recall reaction time and recall accuracy of participants were analyzed using a mixed-design ANOVA, incorporating the factors of 2 (groups: expert, control) × 2 (hand suit categorization: regular, irregular) × 2 (hand rank ordering: regular, irregular). (2) Analysis of the recall of honor cards. Given the significance of honor cards in bridge expertise and the rank ordering rules that consider both their numerical value of the card and HCP value, this experiment further analyzed the recall of crucial cards to elucidate the role played by honor cards in expert memory advantage. 

Post-experiment questionnaire: The post-experiment questionnaires were strategically coded for different tasks. Some participants reported a single strategy, while others mentioned multiple strategies. Therefore, these strategies were aggregated, and the proportion of each mentioned strategy was calculated, specifically referring to the ratio of participants who mentioned a particular strategy within the entire experimental participants.

All data analyses were conducted using SPSS 27.0, with statistical significance set at *p* < 0.05.

### 2.2. Results

#### 2.2.1. Data Organization

In Experiment 1, a total of 107 sets of data were collected. According to the data processing standard, seven sets of data were excluded (one extreme data set, five invalid data sets from participants who did not complete the entire experiment, and one data set with non-compliant responses), accounting for 6.54% of the total data. Finally, there remained 100 sets of usable data remained (50 in the expert group and 50 in the control group). After organizing the data, there were no significant differences between the expert and control groups in gender, age, educational level, intelligence, and accuracy of discrimination reaction in the color discrimination reaction task (*p*s > 0.05). However, there was a significant difference in color discrimination reaction (*t*(98) = 3.54, *p* < 0.001, Cohen’s *d* = 0.71, 95%CI [36.41, 129.04]), with the expert group showing significantly faster color discrimination reaction times compared to those of the control group, so this indicator was used as a covariate in the analysis of correct recognition reaction time.

#### 2.2.2. Recognition Task

(1) Recognition Performance

**Figure 4:**
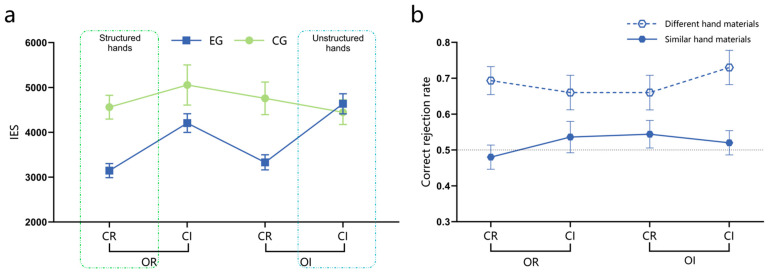
Recognition task-related data figures. (**a**) IES of the expert and control groups under different experimental conditions. (**b**) Correct rejection rates of similar and different hand materials by the expert group under different experimental conditions. Note. EG: expert group; CG: control group; CR: regular suit categorization; CI: irregular suit categorization; OR: regular rank ordering; OI: irregular rank ordering.

The results of the variance analysis for recognition accuracy and the correct recognition reaction time are detailed in the Supplementary Materials, specifically in Table S1 and Figure S1a,b. Since the recognition accuracy and correct recognition reaction time are not consistent, there may be a situation where the longer the reaction time, the higher the accuracy. Therefore, based on Townsend and Ashby’s (1983) speed-accuracy trade-off (IES = RT/ACC, the larger the IES value indicates worse overall performance), a three-factor mixed-design ANOVA was conducted on IES under different experimental conditions. The results showed that both group (95%CI [−1471.84, −279.88], EG < CG) and suit categorization (95%CI [−933.71, −341.28], CR < CI) had significant main effects. The interaction between group and suit categorization was significant, while all other effects were non-significant. Simple effect analysis found that only the IES of the expert group under the condition of regular suit categorization was significantly lower than that under the irregular suit categorization condition (*p* < 0.001, 95%CI [−1601.24, −763.41]), and there was no significant difference between the control group under these two conditions. In addition, under the condition of regular suit categorization, the IES of the expert group was significantly lower than that of the control group (*p* < 0.001, 95%CI [−2023.41, −808.97]). The remaining effects were not significant, as shown in Figure 4a. After using color discrimination reaction time as a covariate, covariance analysis of IES revealed that the interaction between group and suit categorization remained significant (*p* = 0.002), consistent with the aforementioned results.

The memory advantage of the expert group over the control group for structured and unstructured hands was further investigated by conducting independent sample *t*-tests on the IES under the CR-OR and CI-OI conditions. The results showed that experts had significantly lower IES than the control group under the structured hand condition (CR-OR) (*t*(98) = 4.60, *p* < 0.001, Cohen’s *d* = 0.92, 95%CI [−804.41, −2026.01]). However, there was no significant difference between the two groups under the unstructured hand condition (CI-OI) (*t*(98) = −0.53, *p* > 0.05).

(2) The rejection performance of the expert group to unlearned materials

Regardless of whether the unlearned hand materials were similar or different, there were no significant differences in the expert group’s correct rejection rates for these materials across the CR-OR, CR-OI, CI-OR, and CI-OI conditions (*p*s > 0.05). However, across all conditions, the correct rejection rates for different hand materials were significantly higher than those for similar hand materials (*p*s < 0.05). Specifically, the correct rejection rates for similar hand materials did not significantly differ from the random 50% chance level (*p*s > 0.05), while those for different hand materials differed significantly from it (*p*s < 0.01), as shown in Figure 4b.

#### 2.2.3. Recall Task

In terms of recall reaction time, there were significant main effects of group (*F*_(1,98)_ = 13.11, *p* < 0.001, $\eta_{p}^{2}$ = 0.118, 95%CI [−5253.92, −1533.91], EG < CG) and suit categorization (*F*_(1,98)_ = 6.72, *p* = 0.011, $\eta_{p}^{2}$ = 0.064, 95%CI [115.43, 868.80], CR > CI). Additionally, the interaction between group and suit categorization was significant (*F*_(1,98)_ = 8.89, *p* = 0.004, $\eta_{p}^{2}$ = 0.083), as well as the three-way interaction among group, suit categorization, and rank ordering (*F*_(1,98)_ = 5.88, *p* = 0.017, $\eta_{p}^{2}$ = 0.057). Further analysis revealed that experts had a faster recall reaction time in the irregular rank ordering condition compared to the regular rank ordering condition under the condition of the irregular suit categorization (*p* = 0.023, 95%CI [−906.95, −68.69]). Regardless of hand rank ordering, experts’ recall reaction time is faster under the condition of irregular suit categorization than under the condition of regular suit categorization (*p*s < 0.05). Furthermore, experts exhibited faster recall reaction times than the control group across all conditions (*p*s < 0.01). All other effects were non-significant.

**Figure 5:**
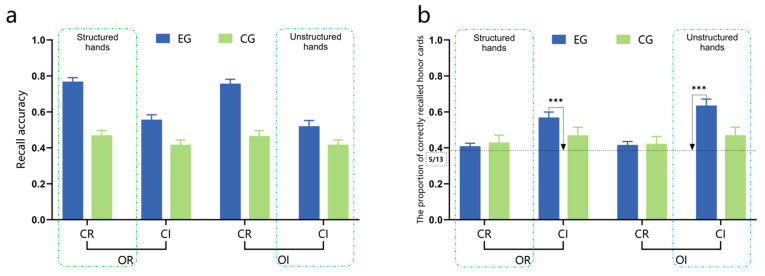
The recall performance of the expert and control groups under different experimental conditions. (**a**) Recall accuracy. (**b**) The proportion of correctly recalled honor cards. Note. *** *p* < 0.001. EG: expert group; CG: control group; CR: regular suit categorization; CI: irregular suit categorization; OR: regular rank ordering; OI: irregular rank ordering.

In terms of recall accuracy, significant main effects were found for group (*F*_(1,98)_ = 38.33, *p* < 0.001, $\eta_{p}^{2}$ = 0.281, 95%CI [0.14, 0.27], EG > CG) and suit categorization (*F*_(1,98)_ = 75.33, *p* < 0.001,$\eta_{p}^{2}$ = 0.435, 95%CI [0.11, 0.17], CR > CI), with a significant interaction effect between group and suit categorization (*F*_(1,98)_ = 30.21, *p* < 0.001, $\eta_{p}^{2}$ = 0.236). Further analysis revealed that regardless of whether the suit categorization was regular or irregular, the recall performance of the expert group was significantly superior to that of the control group (*p*s < 0.01). Additionally, there was a significant effect of suit categorization for both groups (*p*s < 0.05), as illustrated in Figure 5a, while all other effects were non-significant. To analyze the contribution of honor cards to recall accuracy, we calculated the proportion of correctly recalled honor cards for both the expert and control groups under different conditions. This refers to the proportion of honor cards that were recalled correctly out of all the correctly recalled cards. Since each hand in each experimental condition contained an average of five honor cards, if participants’ proportion of correctly recalled honor cards exceeded 5/13, it indicated that they had remembered more honor cards, suggesting the significant role played by these cards in their memorization process. Single-sample *t*-tests were conducted to compare the proportion of correctly recalled honor cards by the expert and control groups under the four hand rule conditions (CR-OR, CR-OI, CI-OR, and CI-OI) with a target proportion of 5/13. The results showed that only in the condition of irregular suit categorization, regardless of the regular or irregular rank ordering, the expert group significantly outperformed with a higher proportion of correctly recalled honor cards than 5/13 (*p*s < 0.001). No significant differences were observed among other conditions (*p*s > 0.05), as shown in Figure 5b.

**Figure 6:**
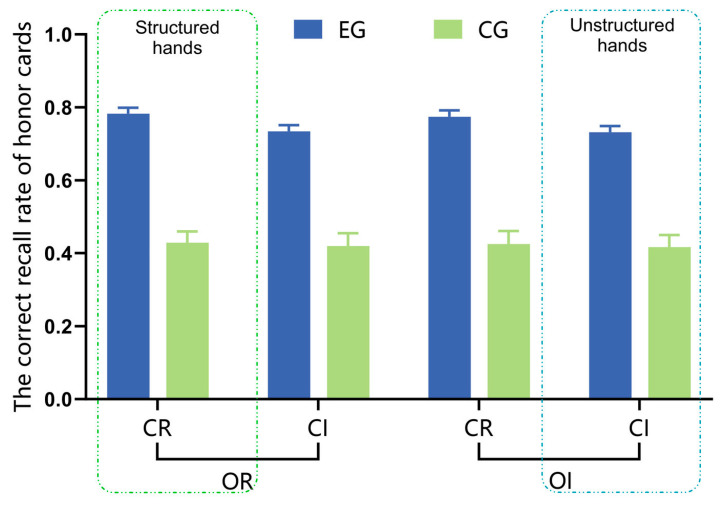
The correct recall rate of honor cards for the expert and the control groups under different experimental conditions. Note. EG: expert group; CG: control group; CR: regular suit categorization; CI: irregular suit categorization; OR: regular rank ordering; OI: irregular rank ordering.

The correct recall rate of honor cards was calculated for the expert group and the control group under different experimental conditions, which refers to the number of correctly recalled honor cards divided by the total number of honor cards presented in each condition. After conducting an analysis of variance, it was found that there were only main effects of group (*F*_(1,98)_ = 88.02, *p* < 0.001, $\eta_{p}^{2}$ = 0.473, 95%CI [0.26, 0.40], EG > CG) and suit categorization (*F*_(1,98)_ = 7.56, *p* = 0.007, $\eta_{p}^{2}$ = 0.072, 95%CI [0.01, 0.05], CR > CI) on the correct recall rate of honor cards, as shown in Figure 6.

#### 2.2.4. Post-Experiment Questionnaire

**Figure 7:**
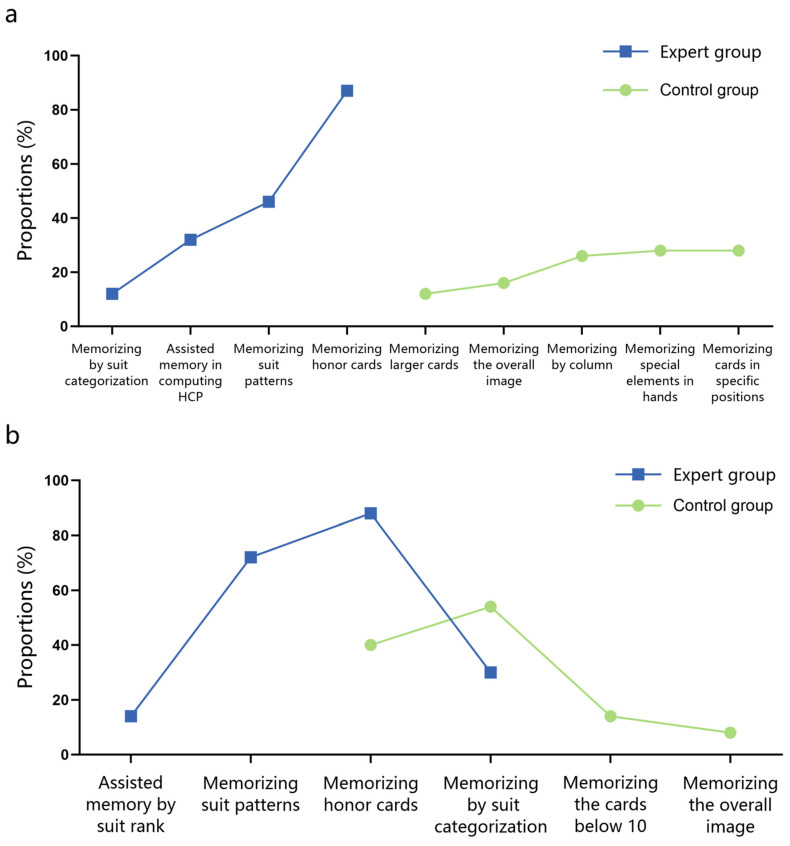
Memory strategies of the expert and control groups under recognition and recall tasks. (**a**) Memory strategies for recognition task. (**b**) Memory strategies for recall task.

In the recognition task, the expert group reported four memory strategies. Among these, 86% of the experts reported employing the strategy of memorizing honor cards, while only 12% reported using the approach of memorizing through suit categorization. In contrast, the control group reported five distinct memory strategies that did not overlap with those identified by the expert group, as detailed in Figure 7a.

During the recall task, both the expert group and the control group reported four distinct strategies, with an overlap observed between the strategy of memorizing honor cards and that of categorizing by suit. Similar to the recognition task, the expert group predominantly employed the strategy of memorizing honor cards (88%), while the control group more frequently utilized the strategy of remembering through suit categorization (54%), as depicted in Figure 7b.

### 2.3. Discussion

The results of Experiment 1 revealed the following: (1) In the recognition task, only under the condition of regular suit categorization, the expert group exhibited a significantly lower IES compared to the control group, indicating their superior memory advantage. A significant difference was observed between the expert and control groups in the structured hand condition (CR-OR), while no significant difference was found in the unstructured hand condition (CI-OI). Upon further comparison of the recognition performance on unlearned materials by the expert group, it was found that they were proficient in effectively distinguishing dissimilar hand materials but struggled to accurately identify similar hand materials with only a change in one honor card’s suit or HCP. (2) In the recall task, irrespective of whether the suit categorization was regular or irregular, the expert group exhibited significantly superior recall performance compared to the control group. The impact of the suit categorization rule was more pronounced among the expert group, as they exhibited a higher accuracy in recalling honor cards under the condition of irregular suit categorization. Across all experimental conditions, the expert group consistently demonstrated a significantly superior correct recall rate for honor cards compared to the control group. Specifically, the correct recall rate for honor cards was notably higher when employing regular suit categorization as opposed to irregular suit categorization.

In both recognition and recall tasks, this research revealed a memory advantage for bridge experts under the structured hand condition, aligning with abundant research on expertise in the field (Corbett et al., 2024; E. T. Smith et al., 2021; Zimmer & Fischer, 2020; Liu, 2019; F. Wang et al., 2016; Bilalić et al., 2010; Schultetus & Charness, 1999; Gobet & Simon, 1996a). However, in the unstructured hand condition, the results diverged: while no significant disparity was found between experts and controls in recognition performance, the expert group exhibited significantly superior recall performance compared to the control group. This discrepancy may be attributed to the differential emphasis on cognitive processes inherent in these two types of memory tasks.

Previous studies commonly manipulated structured hands using a combination of rules, posing challenges in disentangling the underlying factors contributing to expert memory advantages. Expanding on prior research, this research redesigned structured hands and systematically examined the impact of the suit categorization rule and rank ordering rule. The experimental findings revealed that the suit categorization rule exerted a more pronounced influence on experts, irrespective of recognition or recall tasks, whereas the rank ordering rule exhibited no significant impact on either experts or controls. These results imply that the suit categorization rule plays a crucial role in experts’ memory advantage. However, upon analyzing the honor cards containing HCP within the regular rank ordering rule, it was found that irrespective of the experimental conditions, the expert group exhibited a significantly higher accuracy in recalling honor cards compared to the control group. Additionally, there was an interaction impact between the suit categorization and honor cards, indicating that experts exhibited enhanced recall of honor cards when faced with the hands of irregular suit categorization. Most experts reported employing strategies for memorizing honor cards in the post-experimental questionnaire. Collectively, these findings provide compelling evidence for the significant role played by card value (specifically honor cards) in memory processing among experts. 

The findings of Experiment 1 in this research validate that both the suit categorization rule and honor cards rule exert an influence on experts’ memory advantage. However, over years of training, these two rules not only increase experts’ visual familiarity with hand materials but also facilitate their internalization of the rules. Therefore, the findings of Experiment 1 do not provide a definitive explanation for the underlying mechanism through which these two rules impact experts’ memory advantage. In order to address this gap, Experiment 2 was designed to investigate how the specificity information (visual familiarity induced by these rules) and abstract information (abstract knowledge embedded within the rules) influence experts’ memory advantage by manipulating the target objects associated with both suit categorization rule and honor cards rule.

## 3. Experiment 2: The Effects of Suit Categorization Rule and Honor Cards Rule on Memory Performance of Bridge Experts Under Different Objects

### 3.1. Methods

#### 3.1.1. Participants

Using the same pool of expert participants as Experiment 1, data collection for Experiment 2 took place approximately one month later. Due to personal reasons, 7 experts did not participate in Experiment 2, resulting in a total of 45 experts (25 males, 20 females) with an average age of 35.22 years (*SD* = 11.05). All participants had attained at least a high school education level or higher, with 3 high school graduates, 4 associate degree holders, 35 bachelor’s degree holders, and 3 graduate degree holders. The participants’ bridge experience ranged from 10 to 30 years, with an average of 18.31 (*SD* = 5.18) weekly training hours. The total accumulated training time varied between 10,018.29 and 31,307.14 h. While the master points achieved ranged from 250 to 7668 points. According to the “Technical Level Standards for Members of the Chinese Contract Bridge Association” (Chinese Contract Bridge Association, 2023), there were a total of 36 National Masters, 6 Life Masters, and 3 Grand Masters among the participants.

#### 3.1.2. Experimental Design

The experimental design for both recognition and recall tasks in this experiment employed a three-factor within-subject experimental design, consisting of a 2 (suit categorization: regular/CR, irregular/CI) × 2 (honor card objects: unchanged/HU, changed/HC) × 2 (suit objects: unchanged/SU, changed/SC). Here, the condition of unchanged honor card objects refers to honor cards in the hand remaining as A (4 HCP), K (3 HCP), Q (2 HCP), J (1 HCP), and 10 (0 HCP), and the hands in this condition are ranked from largest to smallest. The condition of changed honor card objects pertains to honor cards that have been modified to 1 (1 HCP), 2 (2 HCP), 3 (3 HCP), 4 (4 HCP), and 5 (0 HCP), and the hands in this state are ranked in ascending order. The condition of unchanged suit objects refers to the suits in the hand remaining as spades, hearts, diamonds (red), and clubs (black). And the condition of changed suit objects refers to the suits in the hand changed to black stars, red squares, red triangles, and black circles. The manipulation of suit categorization variables was consistent with Experiment 1. Given that the rule-based abstract knowledge pertaining to honor cards is demonstrated through the association between honor card objects and their respective HCP values, such as A corresponding to 4 HCP or “4” corresponding to 4 HCP, this correspondence directly reflects on the properties of honor card objects. Therefore, in line with this rationale, this research solely manipulated the variable of honor card objects. As the CR-OR and CI-OR conditions in Experiment 1 corresponded to the CR-SU-HU and CI-SU-HU conditions, respectively, and considering that both Experiment 1 and Experiment 2 shared the same pool of participants, data collection for these two experimental conditions was not duplicated in Experiment 2. The dependent variables in this experiment remained consistent with those of Experiment 1. 

#### 3.1.3. Experimental Questionnaire

A self-developed post-experimental questionnaire, identical to the one used in Experiment 1, was used.

#### 3.1.4. Experimental Tasks

The experimental task in this research was conducted following the same procedures as Experiment 1, with the exception of the subsequent modifications.

**Figure 8:**
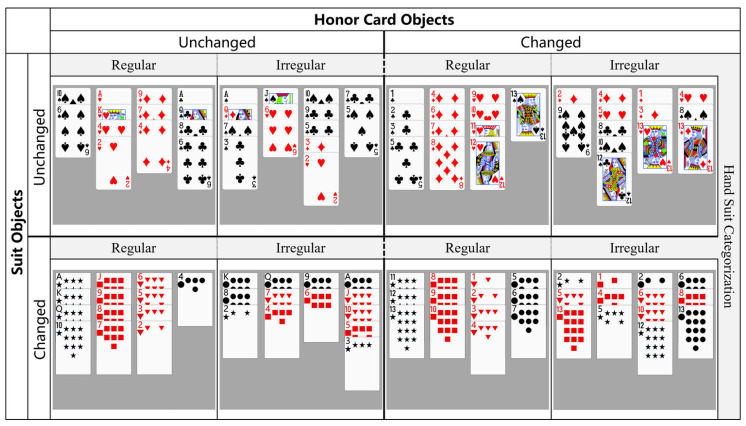
Experiment 2 materials.

Hand materials were created for this research in accordance with the design requirements. Regardless of whether it is the recognition or recall task, the quantities of suits, honor cards (A, K, Q, J, 10, or 1, 2, 3, 4, 5), honor cards with HCP (A, K, Q, J, or 1, 2, 3, 4), and honor cards without HCP (10 or 5) were controlled and balanced across the eight experimental conditions, as well as the HCP of each hand and suit patterns. Specifically, there were no significant differences in the quantities of high-level black suits (spades or stars), high-level red suits (hearts or squares), low-level red suits (diamonds or triangles), and low-level black suits (clubs or circles) under each condition. On average, each hand contains five honor cards in total, with four of them having HCP and one without HCP (10 or 5), resulting in a total of ten HCP. The suit patterns also followed the ten most common suit patterns in bridge. Under the condition of regular suit categorization, the suits in each column are balanced according to the Latin Square. Detailed experimental materials are depicted in Figure 8, and specific parameters for the experimental materials can be found in the Supplementary Materials.

Due to the completion of data collection on regular and irregular suit categorization in Experiment 1, with unchanged suit objects and honor card objects, the remaining six experimental conditions are divided into three runs. These runs include changes in suit objects but unchanged honor card objects with regular or irregular suit categorization (SC-HU-CR/CI), unchanged suit objects but changed honor card objects with regular or irregular suit categorization (SU-HC-CR/CI), and changes in both suit objects and honor card objects with regular or irregular suit categorization (SC-HC-CR/CI). The recognition and recall tasks both encompass these three runs. The settings are consistent among these three runs, except for the experimental materials. The execution order of the runs is balanced between participants using a Latin Square design.

In each run of the recognition task, the learning stage consists of 20 hand materials with regular suit categorization and 20 hand materials with irregular suit categorization; the testing stage consists of 15 learned hand materials and 15 unlearned hand materials. Among the unlearned hand materials, 2/3 of them are similar to the learned hand materials (with only one honor card’s suit or HCP changed), while 1/3 of them are new experimental materials that differ greatly from the learned hand materials. In each recall task run, there are 10 hand materials with regular suit categorization and 10 hand materials with irregular suit categorization in the learning set.

#### 3.1.5. Experimental Procedure

This experiment still uses the TClab online experimental platform, and the experimental program is self-developed. Participants are required to fill out an informed consent form before completing the experimental tasks on a computer. After fully practicing the tasks, they can enter the formal experiment. Once all runs are completed, subjects need to fill out a post-experiment questionnaire.

#### 3.1.6. Data Analysis

The criteria for organizing the data were consistent with those employed in Experiment 1.

The memory performance in both recognition and recall tasks, including measures such as recognition accuracy, correct recognition reaction time, IES, and recall accuracy (specifically the correct recall rate of honor cards), was analyzed using a repeated measures analysis of variance with a 2 (hand suit categorization: regular, irregular) × 2 (honor card objects: unchanged, changed) × 2 (suit objects: unchanged, changed) design.

To explore the role of abstract knowledge within the rules, Experiment 2 also focused on analyzing the following aspects: (1) The role of abstract knowledge in the suit categorization rule was examined by comparing the memory performance of the regular and irregular suit categorization, where both honor card objects and suit objects changed. If a significant difference is found between these two conditions, indicating the presence of a hand suit categorization effect, it suggests that abstract knowledge in the suit categorization rule is effective. (2) The role of abstract knowledge in the honor cards rule was examined by comparing the memory performance of unchanged and changed honor card objects, where the suit categorization is irregular and the suit objects changed. If there is no significant difference between these two, it indicates that abstract knowledge plays a role in the rule of honor cards.

Experiment 2 conducted focused analyses on the following aspects to explore the role of specific visual information: (1) The role of visual information in the suit categorization rule was examined by comparing differences between unchanged and changed suit objects where honor card objects changed, and suit categorization is irregular. If a significant difference exists between these conditions, it indicates that the memory of experts can be influenced by visual familiarity induced by the suit categorization rule. (2) The role of visual information in the honor card rule was examined by comparing differences between unchanged and changed honor card objects where suit categorization is irregular and suit objects changed. If a significant difference exists between these conditions, it indicates that the memory of experts can be influenced by visual familiarity induced by the honor card rule.

### 3.2. Results

#### 3.2.1. Data Organization

A total of forty-five sets of data were collected in Experiment 2, with one set of data being excluded according to the standard (due to invalidity), accounting for 2.22% of the total data. Finally, 44 sets of usable data remained.

#### 3.2.2. Recognition Task

(1) Recognition Performance

**Figure 9:**
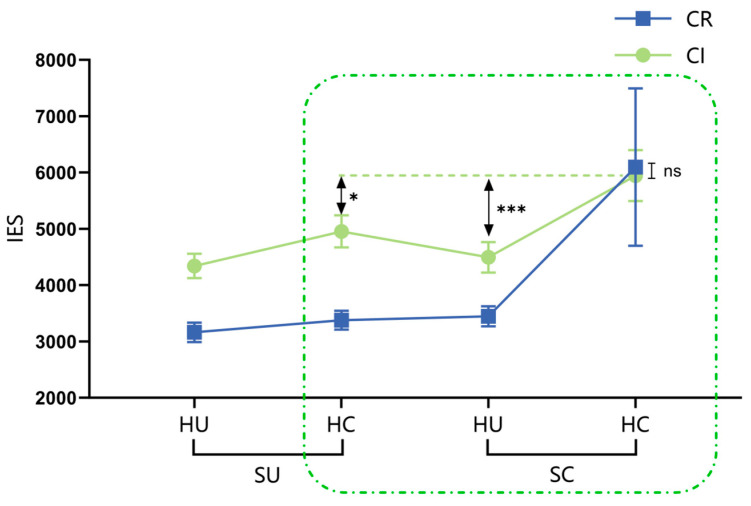
IES of experts under different experimental conditions. Note. * *p* < 0.05; *** *p* < 0.001, “ns” signifies “not significant”. CR: regular suit categorization; CI: irregular suit categorization; HU: unchanged honor card objects; HC: changed honor card objects; SU: unchanged suit objects; SC: changed suit objects.

The results of the variance analysis for recognition accuracy and the correct recognition reaction time are detailed in the Supplementary Materials, specifically in Table S2 and Figure S2a,b. Similarly, a three-factor repeated measures ANOVA was conducted on IES under different experimental conditions. The results showed that the main effects of suit categorization (95%CI [−1700.67, −125.52], CR < CI), honor card objects (95%CI [−1949.60, −514.90], HU < HC), and suit objects (95%CI [−1793.24, −281.01], SU < SC) were all significant. The interaction effect between honor card objects and suit objects was significant. Simple effect analysis revealed that only under the SU-HC condition was the IES significantly lower than under the SC-HC condition (*p* = 0.010, 95% CI [−3253.20, −457.47]). Regardless of whether the suit objects remained unchanged or changed, the IES was significantly lower under the condition of unchanged honor card objects than that of changed honor card objects (*p*s < 0.01), as shown in Figure 9.

**Figure 10:**
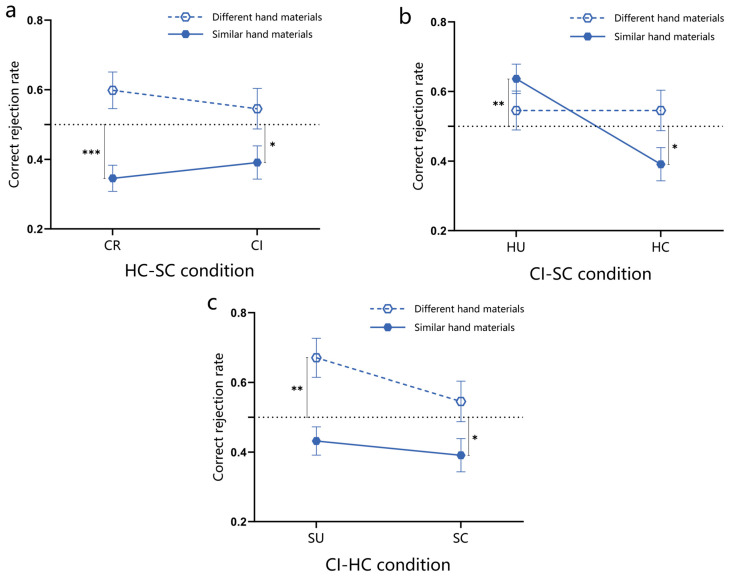
The rejection performance of bridge experts to unlearned materials. (**a**) HC-SC condition. (**b**) CI-SC condition. (**c**) CI-HC condition. Note. * *p* < 0.05; ** *p* < 0.01; *** *p* < 0.001. CR: regular suit categorization; CI: irregular suit categorization; HU: unchanged honor card objects; HC: changed honor card objects; SU: unchanged suit objects; SC: changed suit objects.

Under further analysis of the impact of abstract knowledge and specific visual information in the suit categorization rule and honor cards rule on experts’ memory performance, the results revealed that there was no significant effect of suit categorization under the HC-SC condition, whether in recognition accuracy, correct recognition reaction time, or IES (*p*s > 0.05). Under the CI-SC condition, recognition performance (the above three dependent variables) was significantly lower under the unchanged honor card objects condition than under the changed honor card objects condition (*p*s < 0.05). Additionally, under the CI-HC condition, the IES for unchanged suit objects was significantly lower than for changed suit objects (*p* = 0.014, 95%CI [−1772.53, −210.98]). These results, highlighted within the green frame in Figure 10, indicate that abstract knowledge itself in these two rules did not impact experts’ recognition performance. Instead, the visual familiarity induced by suits and honor cards played a significant role.

(2) The rejection performance of bridge experts to unlearned materials

A repeated measures analysis of variance was conducted under the HC-SC condition, with a 2 (hand materials: similar, different) × 2 (suit categorization: regular, irregular) design. The results showed that the main effect of hand materials was significant (*F*_(1,43)_ = 29.16, *p* < 0.001, $\eta_{p}^{2}$ = 0.404, 95%CI [0.13, 0.28]), and the correct rejection rate of experts for different hand materials was significantly higher than that for similar hand materials. Further analysis compared the experts’ correct rejection rates under the aforementioned conditions with a random baseline of 0.5 and indicated that the correct rejection rates for similar hand materials under the HC-SC condition were significantly lower than 0.5 (*p*s < 0.05). These findings are illustrated in Figure 10a.

A repeated measures analysis of variance was conducted under the CI-SC condition, with a 2 (hand materials: similar, different) × 2 (honor card objects: unchanged, changed) design. The results showed that the main effect of honor card objects was significant (*F*_(1,43)_ = 6.18, *p* = 0.017, $\eta_{p}^{2}$ = 0.126, 95%CI [0.02, 0.22]). Under the condition of unchanged honor card objects, the correct rejection rate was significantly higher than that under the condition of changed honor card objects. The interaction between honor card objects and hand materials was significant (*F*_(1,43)_ = 9.46, *p* = 0.004, $\eta_{p}^{2}$ = 0.180). Simple effect analysis revealed that under the condition of unchanged honor card objects, experts had a significantly higher correct rejection rate compared to the condition of changed honor card objects for similar hand materials (*p* < 0.001, 95%CI [0.13, 0.36]). However, there was no significant difference in the correct rejection rate of experts between the condition of changed and unchanged honor card objects for different hand materials. Further analysis compared the experts’ correct rejection rates under the aforementioned conditions with a random baseline of 0.5. The results indicated that under the CI-HU-SC condition, there was a significant increase in correct rejection rates for similar hand materials compared to 0.5 (*p* = 0.002, 95%CI [0.05, 0.22]). Conversely, under the CI-HC-SC condition, there was a significant decrease in correct rejection rates for similar hand materials compared to 0.5 (*p* = 0.027, 95%CI [−0.21, −0.01]). These findings are illustrated in Figure 10b, suggesting that visual familiarity with honor cards affects the recognition of similar hand materials.

A repeated measures analysis of variance was conducted under the CI-HC condition, with a 2 (hand materials: similar, different) × 2 (suit objects: unchanged, changed) design. The results revealed a significant main effect of hand materials (*F*_(1,43)_ = 18.89, *p* < 0.001, $\eta_{p}^{2}$ = 0.305, 95%CI [0.11, 0.29]), with experts’ correct rejection rates being significantly higher for different hand materials compared to similar hand materials. Further analysis compared the experts’ correct rejection rates under the aforementioned conditions with a random baseline of 0.5. The results showed that under the CI-HC-SU condition, the correct rejection rates for different hand materials were significantly higher than 0.5 (*p* = 0.004, 95%CI [0.06, 0.28]). However, under the CI-HC-SC condition, the correct rejection rates for similar hand materials were significantly lower than 0.5 (*p* = 0.027, 95%CI [−0.21, −0.01]). These findings are illustrated in Figure 10c.

#### 3.2.3. Recall Task

**Figure 11:**
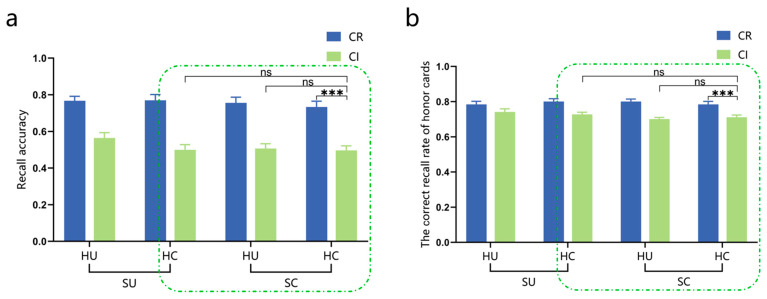
Experts’ recall performance under different experimental conditions. (**a**) Recall accuracy. (**b**) The correct recall rate of honor cards. Note. *** *p* < 0.001, “ns” signifies “not significant”. CR: regular suit categorization; CI: irregular suit categorization; HU: unchanged honor card objects; HC: changed honor card objects; SU: unchanged suit objects; SC: changed suit objects.

The main effects of suit categorization (95%CI [0.19, 0.29], CR > CI), honor card objects (95%CI [0.01, 0.04], HU > HC), and suit objects (95%CI [0.01, 0.05], SU > SC) were all significant in terms of recall accuracy. A significant three-way interaction among suit categorization, honor card objects, and suit objects was also observed. Further analysis revealed that, under the CR-SC and CI-SU conditions, the recall performance of the unchanged honor card objects was superior to that of the changed honor card objects (*p*s < 0.05). Under the CR-HC and CI-HU conditions, the recall performance of the unchanged suit objects was better than that of the changed suit objects (*p*s < 0.05). Regardless of whether the honor card objects and suit objects remained unchanged or changed, the significant effect of the suit categorization was observed (*p*s < 0.001), as illustrated in Figure 11a. 

The main effect of suit categorization on the correct recall rate of honor cards was significant (95%CI [0.05, 0.09], CR > CI), and there was also a significant three-way interaction among suit categorization, honor card objects, and suit objects. The correct recall rate of honor cards with unchanged suit objects was significantly higher than that with changed suit objects under the CI-HU condition (*p* = 0.018, 95%CI [0.05, 0.09]). A significant suit categorization effect was observed regardless of whether the honor card objects and suit objects remained unchanged or changed (*p*s < 0.01), as illustrated in Figure 11b and Table 2.

Further analysis of the impact of abstract knowledge and specific visual information within the rules on expert memory performance reveals a significant effect of suit categorization on both recall accuracy and correct recall rate of honor cards under the HC-SC condition (*p*s < 0.001). However, under the CI-SC condition, whether the honor card objects remain unchanged or are changed does not affect expert memory performance (*p*s > 0.05). The suit objects did not affect expert memory performance under the CI-HC condition, regardless of whether they changed or not (*p*s > 0.05). This suggests that abstract knowledge in both the suit categorization rule and the honor cards rule influences expert memory. These results are depicted within the green frame of Figure 11.

#### 3.2.4. Post-Experimental Questionnaire

**Figure 12:**
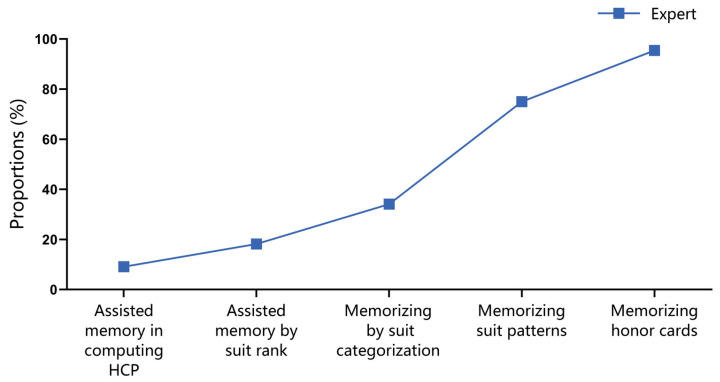
Memory strategies of experts in Experiment 2.

In Experiment 2, experts reported five memory strategies. Consistent with Experiment 1, the majority of experts (95.46%) primarily utilized the strategy of memorizing honor cards, and 34.09% of experts relied on strategies of the suit categorization rule to memorize hands, as shown in Figure 12.

### 3.3. Discussion

This research found the following: (1) In the recognition task, both suit objects and honor card objects collectively influence experts’ recognition performance. The visual familiarity of the expert with the suit objects and the honor card objects significantly influences their recognition performance, but the abstract knowledge within these two rules does not play a role. Experts’ correct rejection rates for unlearned materials revealed that only the honor card objects influenced the correct rejection rates for similar hand materials. (2) In the recall task, suit objects and honor card objects, as well as the suit categorization rule, collectively influence experts’ recall performance. The visual familiarity of rule objects and the abstract knowledge within the rules commonly impact experts’ memory performance in terms of both recall accuracy and correct recall rate of honor cards. 

The present research is consistent with previous findings. A substantial body of the literature suggests that recognition and recall processes exhibit distinct characteristics (Mayas et al., 2024; Montoro & Ruiz, 2022; Craik, 2022; Uner & Roediger, 2022; Du Plessis, 1994; Haist et al., 1992; Loftus, 1971). Recognition tasks primarily involve rapid judgments achieved through the holistic processing of stimulus materials (Mayas et al., 2024; Montoro & Ruiz, 2022; Du Plessis, 1994), wherein visual information plays a pivotal role (Mayas et al., 2024; Sheridan & Reingold, 2017; Brams et al., 2019; Kundel et al., 2007). Consequently, in recognition tasks, the visual familiarity of rule objects influences experts’ performance in recognizing them. In contrast, recall tasks require a more intricate processing of materials, placing emphasis on the memorization and retrieval of all pertinent information (Mayas et al., 2024; Montoro & Ruiz, 2022; Schultetus & Charness, 1999; Gobet & Simon, 1996a). Therefore, both the visual familiarity and the abstract knowledge pertaining to the rules have an impact on experts’ recall performance.

## 4. General Discussion

This research investigates the memory performance of bridge experts and laypersons in relation to different types of hand materials, examining both recall and recognition perspectives. The results revealed that the memory advantage of bridge experts is influenced by both the suit categorization rule and honor cards rule in recognition tasks, with visual familiarity induced by the rules playing a pivotal role. In recall tasks, although consistent phenomena were observed, experts’ memory performance was collectively influenced by their visual familiarity and abstract knowledge of these two rules. Moreover, the memory advantage of bridge experts was not apparent in the recognition task for unstructured hands; however, it was evident in the recall task.

### 4.1. The Underlying Mechanisms of the Memory Superiority in Bridge Experts

This research revealed that bridge experts exhibited superior performance in both recognition and recall tasks compared to laypersons, indicating a significant group effect. These findings suggest that bridge experts possess a memory advantage, and this advantage is influenced by the suit categorization rule and honor cards rule. Research on bridge playing strategies suggests that high-level bridge players rely on continuous calculation and memory of cards’ suits and ranks. The processing information regarding suit and honor cards permeates the entire course of bridge gameplay, and if these pieces of information are effectively utilized by the players, their chances of winning can be significantly increased (Karpin, 1982; Engle & Bukstel, 1978). 

From the perspective of memory recognition, Experiment 1 found that only the suit categorization rule influenced the recognition performance of bridge experts, while the rank ordering rule was not effective, but it is possible that the honor cards rule encompassed within the rank ordering rule might have affected the experts’ memory performance. The results of Experiment 2 further supported this view by showing that changing the objects of these two rules led to a decrease in recognition performance for the experts. This suggests that the visual familiarity resulting from the suit categorization rule and the honor card rule played a significant role in the memory advantage observed in bridge experts, thus supporting the chunking/template theory (Gobet & Clarkson, 2004; Gobet, 1998; Gobet & Simon, 1996b; Chase & Simon, 1973a). Previous studies have suggested that recognition tasks emphasize individuals’ holistic visual memory of the stimulus materials (Mayas et al., 2024; Sheridan & Reingold, 2017; Kundel et al., 2007). This holistic processing mainly relies on individuals’ visual familiarity with the stimulus materials—the higher their familiarity, the better the holistic processing (Sheridan & Reingold, 2017). Analysis of the correct rejection rates for unlearned materials in Experiment 1 found that the bridge experts had significantly higher rates of correct rejection of different hand materials compared to similar hand materials under all conditions, while the correct rejection rate for similar hand materials did not differ significantly from the random baseline. For experts, when comparing learned hand materials to similar unlearned hand materials, although there may be slight differences in details (such as changing one honor card’s suit or HCP), it is difficult to distinguish them holistically. This indirectly suggests that individuals engaged in the holistic processing of visual stimuli during recognition tasks (Mayas et al., 2024; Montoro & Ruiz, 2022; Du Plessis, 1994). To further explore the impact of visual familiarity induced by the suit categorization rule and the honor cards rule on recognition processing, Experiment 2 examined the roles of suit objects, honor card objects, and suit categorization rule under the CI-HC, CI-SC, and HC-SC conditions. The results showed that there was no effect of suit objects and suit categorization rule on the correct rejection rate of unlearned hands in the CI-HC and HC-SC conditions. However, experts’ correct rejection rates for different hand materials were significantly higher than those for similar hand materials. The correct rejection rates for similar hand materials were significantly higher under the CI-SC condition when the honor card objects remained unchanged compared to when they were changed. And there was no significant difference in the correct rejection rates for different hand materials between these conditions. This indicates that the visual familiarity induced by the honor cards rule helps individuals discriminate subtle differences in hands. When the organization of hands does not adhere to the suit categorization rule, experts may focus on remembering honor cards and use them as cues to enhance recognition accuracy, but the visual familiarity induced by the suit categorization rule does not have this effect, suggesting that experts process honor cards more profoundly than suits. In any case, years of bridge training increase individuals’ visual familiarity with hands that conform to the suit categorization rule and the honor cards rule. Structured hand images seen and practiced daily are stored as chunks or templates in long-term memory, thereby exhibiting a memory advantage.

From the perspective of memory recall, Experiment 1 found that both the suit categorization rule and the honor cards rule influenced experts’ accuracy in recalling. When the suit categorization was irregular, experts would recall more honor cards. Experiment 2 further demonstrated the interactions among suit objects, honor card objects, and the suit categorization rule, collectively affecting experts’ recall performance. Even with visually less familiar hand materials like HC-SC and CI-SC conditions, experts were able to utilize abstract knowledge of these rules to complete recall tasks. These findings indicate that the visual familiarity induced by these rules and the abstract knowledge of the rules impact experts’ memory performance, thereby supporting both chunking/template theory and SEEK theory. Unlike recognition tasks, recall tasks require more detailed processing of materials, emphasizing the memorization and retrieval of all information (Mayas et al., 2024; Montoro & Ruiz, 2022; Schultetus & Charness, 1999; Gobet & Simon, 1996a). Years of bridge training enable experts not only to form chunks of structured hand images but also to acquire substantial, highly conceptualized knowledge (Gobet & Waters, 2023; Chassy & Gobet, 2011), thereby exhibiting stable memory advantages across different types of hand materials. Given that recall tasks closely mirror the real memory processes of bridge players regarding their hands (Engle & Bukstel, 1978), findings from these tasks may better reflect the underlying mechanisms of memory superiority among bridge experts. 

### 4.2. The Transfer of Memory Advantage in Bridge Experts 

This research found that bridge experts consistently demonstrate memory advantages in both recall and recognition tasks when dealing with structured hand materials. This consistency aligns with previous research (Charness, 1979; Engle & Bukstel, 1978), indicating the significant role of the suit categorization rule and the honor cards rule in the memory superiority of experts. However, inconsistent findings exist regarding the unstructured hands. Charness (1979) found no significant difference in memory performance between bridge experts and novices on unstructured hands. In this research, during the recall task of Experiment 1, bridge experts exhibited significantly higher accuracy in recalling unstructured hands compared to laypersons, but this phenomenon was not observed in recognition tasks. As mentioned earlier, recognition tasks emphasize the holistic processing of visual stimuli (Mayas et al., 2024; Montoro & Ruiz, 2022; Du Plessis, 1994), where unstructured hands may differ significantly from the visually familiar stimuli based on the rules formed by experts, thereby not showing memory advantages in recognition tasks. In contrast, recall tasks require the processing of specific stimulus information involving the memorization and retrieval of all hand details (Mayas et al., 2024; Montoro & Ruiz, 2022; Schultetus & Charness, 1999; Gobet & Simon, 1996a). Both the visual familiarity and abstract knowledge within the suit categorization rule and the honor cards rule influence experts’ recall. Therefore, for visually unfamiliar unstructured hands, experts can efficiently rely on abstract knowledge derived from learned rules to enhance memory performance, thus demonstrating memory advantages in recall tasks.

Extensive research has demonstrated that bridge training can enhance cognitive performance within the domain. For example, the longer the duration of general bridge players’ training, the better their cognitive performance in bidding (Charness, 1987, 1983), card play (Charness, 1979; Engle & Bukstel, 1978), and memory for hands (Engle & Bukstel, 1978). The present research demonstrates that memory advantages for unstructured hands among experts may vary across different memory tasks despite both structured and unstructured hands being domain-specific materials. Therefore, the impact of bridge training on cognitive performance within the domain cannot be generalized and requires a deeper understanding. The findings of this research suggest that while years of training enable bridge experts to develop visual familiarity with hands based on the suit categorization rule and the honor cards rule, this familiarity is more susceptible to influences from factors such as hand organization (structured vs. unstructured) and the type of cognitive task (recognition vs. recall). However, the internalization of abstract knowledge underlying these rules is less affected by these factors for experts. Similar findings have been observed in the domain of chess. For instance, Gong (2015) explored the memory advantages of chess experts and found that, with chess materials presented for a short duration, participants primarily relied on visual processing to complete the task. Under such conditions, the memory advantage of chess experts for authentic chess configurations was significantly greater than that of novices, whereas no significant difference was observed between the two groups for random chess configurations. However, with chess materials presented for longer durations, participants were able to process the chess configurations in greater detail, resulting in significant differences in memory performance between chess experts and novices across all types of chess configurations.

The internalization of abstract knowledge regarding the rules for suit categorization and honor cards by bridge experts not only enhances memory for domain-specific hand materials but also facilitates the transfer of this knowledge to memory for domain-external hand materials. In Experiment 2 of this research, we created domain-external hand materials with low visual familiarity by altering the suit objects and honor card objects in order to investigate whether the abstract knowledge of these rules could improve experts’ recall of domain-external hand materials. The results showed that experts had higher recall accuracy under the regular suit categorization condition compared to the irregular suit categorization condition when hand materials had both changed suit objects and honor card objects. There was no significant difference in recall accuracy between unchanged and changed honor card object condition for hand materials with changed suit objects and irregular suit categorization. These findings indicate that the internalization of abstract knowledge by experts can enhance memory for both domain-specific and domain-external hand materials. In other words, the memory enhancement effect of bridge training is not limited to specific domains but also extends to external contexts, demonstrating a near-transfer role. Similar near-transfer phenomena have been observed in the field of chess research. For instance, a study conducted by E. T. Smith et al. (2021) found that the spatial position memory and discrimination abilities developed by chess experts not only manifested in materials related to chess but also transferred to unfamiliar stimuli, where they exhibited superior performance compared to laypersons.

Therefore, it is clear that extensive bridge training facilitates the acquisition and internalization of relevant rules, enabling individuals to utilize this rule-based knowledge to improve their memory for both domain-specific and domain-external stimuli.

### 4.3. Practical Application Value

This study reveals the significant role of the suit categorization rule and honor cards rule in enhancing the memory capabilities of bridge experts. By deeply exploring how these rules impact memory performance, we can systematically integrate these strategies into training programs, significantly boosting competitive bridge players’ memory and strategic thinking abilities. Specifically, personalized coaching plans can leverage insights from established memory performance metrics to tailor training that optimizes players’ skills in visual recognition and abstract rule application. The training of bridge players requires not only extensive practice but, more importantly, a deep understanding of the rules and knowledge of bridge. Moreover, these research outcomes provide a robust foundation for developing more advanced bridge AI technologies, potentially leading to algorithms that more effectively emulate human strategic thinking in bridge—a complex challenge for AI development. These findings not only help bridge coaches design more effective training programs, targeting memory and strategy use enhancements, but they might also enhance algorithmic performance in simulations, thus elevating competitive bridge training and gameplay to new heights. By harnessing these strategies, players can improve their overall performance and strategic capabilities within the game.

### 4.4. Limitations and Future Prospects

Due to the specificity of expertise research, this research has some limitations. Firstly, the control group in this study was comprised of laypersons who were unfamiliar with bridge, as opposed to novice players. These individuals were merely acquainted with playing cards but lacked knowledge of bridge rules. Consequently, the results obtained could only assess the impact of the presence or absence of bridge rules on memory advantage rather than evaluating the extent of mastery over bridge rules. Further research is required to explore the relationship between the acquisition of bridge skills and memory performance. Secondly, there was a significant variation in the technical proficiency of the participating experts. Despite meeting the recruitment criteria, their technical levels ranged from world-class bridge Grand Masters to National Masters, which could have impacted the experimental outcomes. In addition, in the recall tasks of this research, participants were instructed to memorize cards ranked lower than 10 as “X”, which might have compromised the internal validity of the experiment and potentially influenced the results. Future research could develop experimental tools that closely simulate real bridge-playing scenarios. Finally, this study primarily investigates the points of contention between chunking, template theory, and SEEK theory, focusing on the examination of specific and abstract information. However, it remains unclear whether the combined use of this information relates to the retrieval structures mentioned in the LTWM theory. Future research should delve into this aspect.

Given the current limitations, future research could expand in several directions. Firstly, while this research explored the near transferability of expert memory advantages, future investigations could examine whether bridge experts maintain their memory superiority when dealing with materials outside their domain from a far transfer perspective. Secondly, by integrating neuroimaging techniques such as fMRI and fNIRS, a more in-depth examination of the neural mechanisms behind the memory advantages of bridge experts can be conducted. This approach would elucidate the intrinsic mechanisms of how bridge rules contribute to memory advantages in experts and the neural plasticity changes induced by long-term training. Finally, considering the potential cognitive benefits of bridge training, future research could explore its potential application in preserving cognitive aging and preventing dementia. This would involve evaluating the practical utility of these activities as intervention measures and providing new strategies for maintaining human cognitive health. 

## 5. Conclusions

In summary, this research yields the following conclusions based on two experiments: (1) In recognition tasks, both the suit categorization rule and the honor cards rule significantly contribute to the memory advantage of bridge experts, with the visual familiarity engendered by these rules serving as a pivotal factor. (2) In recall tasks, these two rules similarly influence the recall performance of bridge experts, with both visual familiarity and abstract knowledge elicited by the rules jointly shaping their memory performance. The aforementioned findings demonstrate that the memory advantage possessed by bridge experts arises from the combined effects of visual information and abstract knowledge. These results concurrently support the chunking/template theory and the SEEK theory, providing a more integrated understanding of the intrinsic mechanisms behind the memory advantages of bridge experts. The findings of this study can further guide the training of bridge players and offer recommendations for preventing cognitive aging and related educational programs, demonstrating that prolonged practice in a specific domain can facilitate the near transfer of relevant knowledge.

## Figures and Tables

**Table 1:** Demographic information of the expert group and control group.

	Gender		Educational Background				Age	Intelligence
	Male	Female	High School	Junior College	Undergraduate	Master		
Expert Group	31	21	4	6	39	3	36.12 ± 12.07	45.57 ± 8.00
Control Group	32	23	5	6	39	5	37.09 ± 12.51	44.54 ± 8.17
*t*/*χ*^2^	0.02		0.53				0.41	−0.64

Note. In this table, “intelligence” refers to the score obtained by participants on the Raven’s Progressive Matrices test within a 15-minute period.

**Table 2:** Comparison results of experts’ recall performance under different experimental conditions.

	Recall Accuracy		The Correct Recall Rate of Honor Cards	
	*F*	$\eta_{p}^{2}$	*F*	$\eta_{p}^{2}$
Suit categorization	85.52 ***	0.665	56.21 ***	0.567
Honor card objects	6.54 *	0.132	0.01	0.000
Suit objects	7.90 **	0.155	3.42	0.074
Suit categorization × Honor card objects	3.36	0.072	0.02	0.000
Suit categorization × Suit objects	0.10	0.002	3.77	0.081
Honor card objects × Suit objects	0.73	0.017	0.07	0.002
Suit categorization × Honor card objects × Suit objects	8.10 **	0.158	5.29 *	0.109

Note. * *p* < 0.05; ** *p* < 0.01; *** *p* < 0.001.

## Data Availability

If data and codes are required, they can be obtained by contacting the corresponding author or the first author.

## Supplementary Materials

The following supporting information can be downloaded at: https://www.mdpi.com/article/10.3390/bs15020125/s1.
