# Supplementary material for: Cognitive Mechanisms Underlying Memory Advantages in Bridge Experts: Based on Suit Categorization and Honor Card Rules
Source: Behav Sci (Basel). 2025 Jan 24;15(2):125. doi: 10.3390/bs15020125 (PMC11852315; doi:10.3390/bs15020125)
Supplement: Supplementary file 1 [file behavsci-15-00125-s001.zip › Supplementary Material _Results.pdf]

1 Experiment 1 Recognition Task

The results of the variance analysis of recognition accuracy and correct recognition reaction time are shown in Table S1 and Figure S1 (a), (b).

Table S1 Comparison of Recognition Performance Between the Expert and Control Groups Under Different Experimental Conditions

	Recognition accuracy		Correct recognition reaction time	
	F	η_p^2	*F*	η_p^2
Group	51.46***	0.344	0.20	0.002
Suit categorization	23.63***	0.194	9.89**	0.093
Rank ordering	0.041	0.000	0.54	0.006
Group × Suit categorization	17.98***	0.155	10.33**	0.096
Group × Rank ordering	4.06*	0.040	0.02	0.000
Suit categorization × Rank ordering	0.39	0.004	0.01	0.000
Group × Suit categorization × Rank ordering	0.39	0.004	0.37	0.004

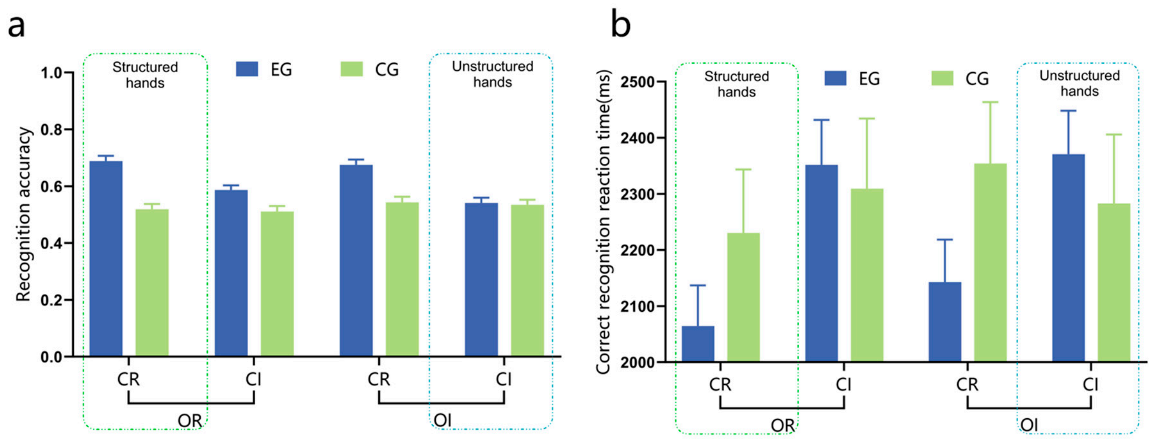

Figure S1 Recognition Performance of the Expert and Control Groups Under Different

Experimental Conditions. (a) Recognition accuracy. (b) Correct recognition reaction time(ms). *Note.* EG: Expert group; CG: Control group; CR: Regular suit categorization; CI: Irregular suit categorization; OR: Regular rank ordering; OI: Irregular rank ordering.

2 Experiment 2 Recognition Task

The results of the variance analysis of recognition accuracy and correct recognition reaction time are presented in Table S2 and Figure S2 (a) and (b).

Table S2 Comparison Results of Experts' Recognition Performance Under Different Experimental Conditions

	Recognition accuracy		Correct recognition reaction time	
	F	η_p^2	F	η_p^2
Suit categorization	62.71***	0.593	18.54***	0.301
Honor card objects	40.06***	0.482	4.96*	0.103
Suit objects	34.16***	0.443	0.63	0.014
Suit categorization × Honor card objects	0.21	0.005	1.92	0.043
Suit categorization × Suit objects	4.87*	0.102	12.07**	0.219
Honor card objects × Suit objects	14.90***	0.257	0.91	0.021
Suit categorization × Honor card objects × Suit objects	6.51*	0.132	1.31	0.030

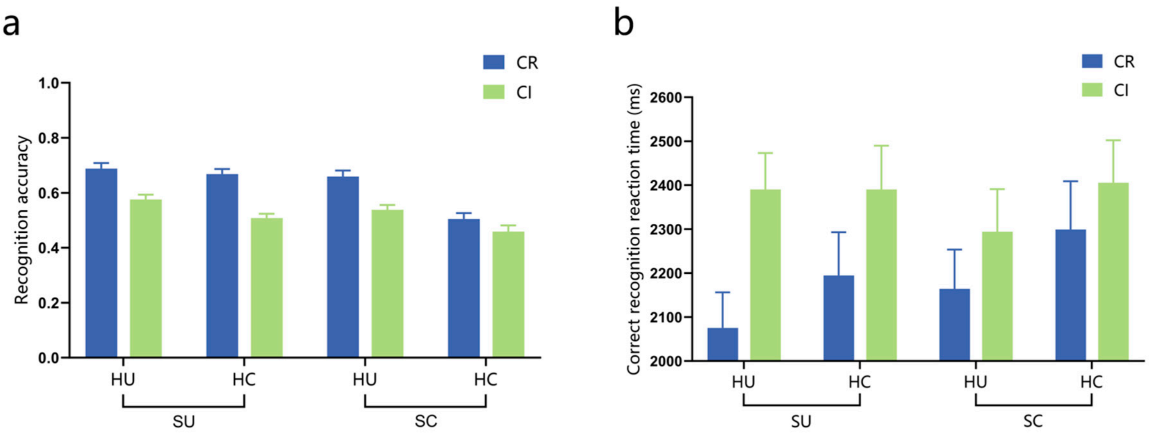

Figure S2 Expert Recognition Performance Under Different Experimental Conditions. (a) Recognition accuracy. (b) Correct recognition reaction time(ms). *Note.* CR: Regular suit categorization; CI: Irregular suit categorization; HU: Unchanged honor card objects; HC: Changed honor card objects; SU: Unchanged suit objects; SC: Changed suit objects.
